# Vermin Vastness

**Published:** 1884-10

**Authors:** 


### FEMININE FASTNESS.

THE AUTHOR of the "Heir of Redclyffe." Miss Yonge, writes thus plainly concerning the association of women and tobacco: "I know I shall offend many of my readers by saying that I think men have done much to lower the tone of refinement in women, in making them submit to smoking Forty or fifty years ago, the gentlemen I knew best (officers in the army, some of them) would have no more thought of accustoming their wives, daughters, or sisters, to the smell of smoke than they would to the atmosphere of a public house. They would have thought that something of the woman's grace was lost by treating her with the disregard thus implied, and that they failed in respect to her sex. Most gentlemen were of this mind; they seldom smoked themselves, and when their sons took up the practice, forbade it in the house, and were much displeased if they saw it done before their daughters. Girls, however, are apt to take the side of their brothers when they think them deprived of a harmless pleasure. Fun did something, and so did the pleasure and honor of being with a brother, and the young men themselves viewed the parental dislike as old fogyism, the feminine distaste as simple fidget and selfishness. I have even seen it argued that smoking is no more selfish than tea drinking—as if tea poisoned the sweet air around it, or left fumes in rooms and clothes. I do not say the sisters were always wrong. It is better to put self aside, than to drive away and lose, a brother's confidence; but I do say that the whole tone between man and woman is lower than it was in those days, and that the habitual self-indulgence in a free-and-easy custom, hardly respectful, cannot but have assisted in this.

And as to the custom creeping in, of girls enjoying cigarettes—a thing begun in fastness and fun, and excused by the customs of foreigners—it is one of the readiest ways of unsexing themselves, and losing all the reverence due to womanhood; which reverence is a greater benefit to man than even to woman.

After this I need hardly say what I think of the practice of going and sitting with men in their smoking-room. We may say we are at home with them and can trust them. I hope we can, but to follow them into their own peculiar haunts is not fit for any woman or girl, and if she does it in thoughtlessness at first, she will either have to draw back or will have the true charm and grace of her sex spoilt. It is not a boon companion that a man wants, it is something to call out his higher feelings of respect and honor.

